# The Relationship Between Loneliness and Psychological Adjustment: Validation of the Italian Version of the Interpersonal Acceptance–Rejection Loneliness Scale

**DOI:** 10.3389/fpsyg.2021.655410

**Published:** 2021-06-03

**Authors:** Vincenzo Paolo Senese, Carla Nasti, Francesca Mottola, Ida Sergi, Rita Massaro, Augusto Gnisci

**Affiliations:** Department of Psychology, University of Campania “Luigi Vanvitelli”, Caserta, Italy

**Keywords:** loneliness, psychological adjustment, Interpersonal Acceptance-Rejection Loneliness Scale, measurement invariance, validity

## Abstract

In recent literature, many researchers have highlighted that the feeling of loneliness can be considered a sign of relevant distress with short- and long-term consequences on the health of people who needs to be appropriately monitored and treated. In this view, the Interpersonal Acceptance–Rejection Loneliness Scale (IPARLS) has been developed to evaluate the subjective feelings and distress related to interpersonal loneliness and to overcome the limits of the previous scales; however, its psychometric characteristics have not yet been fully investigated. Starting from these considerations, two studies have been conducted. The first study, involving 688 adults (19–69 years; 51% females), assessed dimensionality, reliability, and validity of and tested the measurement invariance (MI) of the Italian version of the IPARLS. The second study, involving 205 adults (20–69 years; 51% females), investigated the relationship between loneliness and psychological adjustment. Results confirmed the unidimensionality of the scale and showed that it is reliable, valid, and fully invariant as a function of age and gender. Moreover, data showed a strong association between perceived loneliness and psychological adjustment. The IPARLS is confirmed to be a valid and reliable measure to investigate loneliness in the life cycle from clinical and research perspectives.

## Introduction

In the scientific literature, it is generally assumed and accepted that human beings have an innate predisposition to socialization (Bowlby, 1973; Rohner, 2019; Leary, 2021). Indeed, since after birth and for many years, humans have a need to associate with others to survive because they are not self-sufficient (Bornstein, 2016). The need to be linked to others, or belong to a social community, persists in all main steps of the life cycle. During adolescence, individuals need to feel being part of a social group that supports and accepts them to develop their individuality, psychological adjustment, and behavior (Sani, 2012; Senese et al., 2019); during adulthood, they need others to establish lasting intimate relationships (Arnett, 2007; Senese et al., 2020a); whereas during the old age, the need to belong and to have social relationships becomes even stronger and important because of aging and the loss of self-sufficiency (Fingerman and Pitzer, 2007; Rook and Charles, 2017). However, not only establishing interpersonal relationships but also their quality has an important effect on individuals (Caccioppo and Caccioppo, 2018). Good social relationships, that is social relationships perceived as being emotionally positive or supportive or based on trust, love, warmth, and interpersonal acceptance, are positively associated with well-being (Atkinson et al., 2020). On the contrary, when individuals perceive their main interpersonal relationships as inadequate and unsatisfying, based on rejection and neglect, they feel an unpleasant sensation called loneliness (Peplau and Perlman, 1982; Russell et al., 2012; Mund and Johnson, 2021). A theoretical framework that emphasizes the impact of the subjective perception of the quality of interpersonal relationships throughout the life cycle on psychological well-being is the Interpersonal Acceptance–Rejection Theory (IPARTheory; Rohner, 2019). According to this model, when individuals cannot satisfy their need to be loved or feel accepted by others, in particular from parents, they develop a psychological condition described as “Acceptance–Rejection Syndrome” (Rohner, 2004); this is associated with an increased likelihood of experiencing psychological problems, such as depression, substance abuse, fear of intimacy, and loneliness (Rohner and Lansford, 2017). Therefore, in this model, loneliness is considered as one of the possible consequences of interpersonal rejection. Weiss (1973) differentiated between emotional and social loneliness. Emotional loneliness is defined as the perception of inadequate interpersonal relationships, while social loneliness is the lack of supportive social relationships. It has been shown that the emotional component predicts life satisfaction more than social loneliness and that the absence of intimate close relationships is more distressing than the lack of social relationships (Salimi, 2011; O'Súilleabháin et al., 2019). Similarly, van Tilburg (2020) introduced the concept of existential loneliness as a lack of intimate, personal, and close ties with others, highlighting that this would lead to a reduction of the meaning of life and of problems related to psychological adjustment.

There are a number of studies that have highlighted that loneliness influences the daily life and the general life satisfaction of individuals who experience it (Mellor et al., 2008; Demeter and Rad, 2020) and that individuals distressed by loneliness have an increased likelihood of psychological and health problems than individuals with satisfactory interpersonal relationships (Heinrich and Gullone, 2006; American Psychiatric Association, 2013; Richardson et al., 2017; Menec et al., 2020; Wang et al., 2020). In particular, loneliness has been linked to a variety of negative outcomes, such as the risk of sleep disorders (Griffin et al., 2020), eating disorders (Fox, 2020), Internet addiction (Mazuz and Yom-Tov, 2020), alcohol consumption, gambling (Savolainen et al., 2020), cognitive decline (Sutin et al., 2020), depression, suicide (Martín María et al., 2021), and psychotic symptoms (Ludwig et al., 2020). Researchers investigating the role of gender and age as possible vulnerability factors found mixed results (Barreto et al., 2020; Shovestul et al., 2020). A recent meta-analysis (Maes et al., 2019) carried out to investigate gender differences in loneliness across the lifespan showed that the gender differences were small and that the effect was moderated by age, sampling area heterogeneity, and year of publication. Moreover, the authors highlighted that, in very scarce cases, the considered measures were preliminarily tested for their invariance across gender and age, thus threatening the validity of the results.

According to the Evolutionary Theory of Loneliness (Caccioppo and Caccioppo, 2018), the feeling of loneliness represents an alert signal that has an adaptive value because it should stimulate the brain to initiate a set of correlated behavioral, neural, hormonal, and cellular adjustments aimed at mitigating this unpleasant condition and encouraging the formation of beneficial social relationships. However, individuals are not always successful in improving the quality of their interpersonal relationships. This happens because loneliness can trigger feelings of insecurity, can set individuals in a defensive and hypervigilant position toward others and alter their ability to regulate emotions, increasing anger, avoidance, distrust, hostility, and a negative view of social experiences (Baran et al., 2015; Segel-Karpas and Ayalon, 2020). As a result, the feeling of loneliness can trigger a negative spiral that risks causing social withdrawal or conflicts in social relationships rather than reduce them (Wielinga et al., 2021).

Based on empirical evidence, loneliness can be considered a risk indicator of the physical and mental well-being of the individual or of the life satisfaction of the individual, therefore it is important to have valid and reliable measures of this dimension that allow for the monitoring of the feeling of loneliness across the life cycle. Indeed, there are phases of life, such as old age in particular, where recognizing the presence of high levels of loneliness can be particularly useful to promote targeted interventions to reduce the associated distress and to verify their effectiveness. For example, some recent uses of robotics go in this direction. Indeed, several researchers have pointed out how artificial cognitive systems can be used to increase the well-being of the elderly (Esposito et al., 2014; Baranyi et al., 2015). It seems that the interaction between humans and anthropomorphic social agents or robots (Li et al., 2020) has a positive effect on the feeling of loneliness (Lee et al., 2006), especially for people more inclined to loneliness (Gallego-Perez et al., 2013; Gross et al., 2015).

In the international literature, the most used scales designed to measure loneliness are: (a) the De Jong Gierveld Loneliness Scale (DJG-LS; De Jong-Gierveld and Kamphuls, 1985) and (b) the University of California Los Angeles (UCLA) Loneliness Scale Version 3 (UCLA-LS-III; Russell, 1996). The DJG-LS is a 11-item scale measuring both social and emotional loneliness, according to the loneliness distinction of Weiss (Weiss, 1973). For each item, individuals are asked to indicate how much they agree with a sentence reflecting feelings of loneliness (e.g., “I experience a general sense of emptiness” or “There are many people I can trust completely”) on a 5-point Likert scale (1 = “yes!”; 2 = “yes”; 3 = “more or less”; 4 = “no”; 5 = “no!”). The scale had good reliability and validity (De Jong Gierveld and Van Tilburg, 2006). Moreover, the researchers also tested a 6-item version of the scale, showing that the shortened version of the scales had the same psychometric properties as the original scales (De Jong Gierveld and Van Tilburg, 2006). The 11-item scale was translated into Italian and was used to investigate loneliness in a sample of adults aging 55–89 years (Van Tilburg et al., 1998), but the psychometric characteristics of the Italian version of 11-item and 6-item scales were not directly investigated.

The UCLA-LS-III is a 20-item scale measuring social isolation sensation (Russell, 1996). For each item, individuals are asked to rate their social isolation (e.g., “How often do you feel part of a group of friends?”) on a 4-point Likert-type scale (from 1 = “never” to 4 = “always”). The scale had good reliability and validity (Russell, 1996). Hughes et al. (2004) developed a 3-item version of the UCLA-LS-III, demonstrating that the shortened version has the same psychometric properties as the original scale. The UCLA-LS-III was adapted to Italian and was used to measure loneliness in a sample of 350 university students (Boffo et al., 2012), but the psychometric characteristics of the Italian version were not directly investigated.

Although these scales are widely used, some researchers (see Rohner and Molaver, 2015) have highlighted some limitations. Particularly, Rohner and Molaver (2015) argued that UCLA-LS-III measures social isolation rather than the feeling of loneliness, and that both UCLA-LS-III and DJG-LS scales tend to assess the perception of loneliness in a more objective way, without considering adequately the psychological distress associated with loneliness. Starting from the assumption that subjective feelings about interpersonal relationships are more relevant than the objective richness of the social network (Rohner, 2019) and from IPARTheory, Rohner and Molaver (2015) have developed a new self-report scale in an attempt to overcome the limitations of the previous scales: the Interpersonal Acceptance and Rejection Scale of Loneliness (IPARLS). IPARLS is a 15-item scale developed to measure the subjective feelings about the psychological distress associated with loneliness in an interpersonal perspective (e.g., “I feel badly because I am isolated from others” or “I am distracted by feelings of loneliness”) on a 5-point Likert scale (from 1 = “never true” to 5 = “always true”). The operationalization of the construct is based on the theoretical model of the IPARTheory (Rohner, 2019) that emphasizes the impact of subjective perceptions of the quality of interpersonal relationships on well-being. Recently, the IPARLS was adapted into four languages (Dutch, Kurdish, Italian, and Urdu) and was used to explore the association between the remembrances of parental acceptance–rejection in childhood, psychological maladjustment of adults, and the level of loneliness in five nations (Rohner et al., 2020). The research showed that the scale has adequate psychometric characteristics in each version; however, the latent structure investigations and the validity analysis were not reported.

Taking into consideration that the loneliness scales available in Italian do not provide complete indications on the psychometric properties, that in no case the invariance of the measure across gender and age has been verified, that the emotional and interpersonal component of loneliness is the one that most influences the well-being and adaptation of individuals, and that, recently, the IPARLS has been developed to capture these specific aspects of the construct, the aims of this study are classified into two: (1) investigating the psychometric properties of the Italian version of the IPARLS and (2) investigating the relationship between loneliness distress and psychological adjustment. To this aim, two independent studies were carried out. In the first study (Study 1), the Italian version of the IPARLS was administered to a sample of 688 adults (19–69 years; 51% females) to assess dimensionality, reliability, and validity and to test the measurement invariance (MI) of the scale across gender and age. In the second study (Study 2), the Italian IPARLS was administered in conjunction with the Adult Personality Assessment Questionnaire short-form (PAQ-SF) to a sample of 205 adults (20–69 years; 51% females) to investigate the relationship between loneliness and the psychological well-being.

## Study 1

This study aimed to examine the main psychometric characteristics of the Italian version of the IPARLS (Rohner and Molaver, 2015). For this purpose, a sample of Italian adults was administered the scale, and its psychometric characteristics were studied. Particularly, the reliability, the latent dimensionality, and the MI of the scale across age and gender were investigated. These latter invariance factors have been considered as they are most frequently considered as vulnerability factors (Maes et al., 2019; Barreto et al., 2020; Shovestul et al., 2020) associated with loneliness. In addition, to evaluate the construct validity of the scale, a random subsample of participants completed the IPARLS in combination with the 6-Item DJG-LS (De Jong Gierveld and Van Tilburg, 2006), as a convergent measure of loneliness, or the Lubben Social Network scale (LSNS) (Lubben et al., 2006), as an objective measure of social networks for the discriminant validity. In line with the literature, we expected that IPARLS would have good psychometric characteristics, that IPARLS scores would be more related to the emotional dimension than the social loneliness dimension of the DJG-LS, and that the perception of loneliness would be weakly associated with or would be independent of the richness of the social network. Finally, age and gender differences in IPARLS scores were investigated to verify their association with loneliness distress and to suggest possible expected scores to use in the clinical practice. In line with the previous studies, we expected that there would be more loneliness distress in males than females if the difference was significant and that loneliness would be negatively associated with age (Maes et al., 2019; Barreto et al., 2020; Shovestul et al., 2020).

### Methods

#### Participants

A sample of 688 adults (19–69 years; *M* age = 36.4; *SD* = 14.8), 351 females (51%) and 337 males (49%), was recruited by convenience sampling from different cities of the Campania region (southern Italy). The sample was heterogeneous in terms of the educational level, which ranged from “less than middle school” to “college and above” (Mdn = “partial college, at least 1 year of specialized training”), and the occupational level, which ranged from “no regular occupation” to “higher executive, proprietor of large businesses, major professional, and others” (Mdn = “smaller business owners, skilled manual laborers, craftsmen, tenant farmers, and others”). The considered measures were administered in-person and in a paper-and-pencil format. The average time to complete the protocol was about 13 min. Data were collected in conformity with the Declaration of Helsinki and the local Ethics Committee requirements. All participants signed a written informed consent before starting data collection.

#### Procedure and Measures

Participants completed a protocol consisting of three sections: (1) the sociodemographic form; (2) the IPARLS; and (3) the criterion measure (the DJG-LS or the LSNS).

##### Sociodemographics

All participants completed a questionnaire to collect socio-demographic information (age, gender, educational level, and so on).

##### Interpersonal Acceptance–Rejection Loneliness Scale

The Italian version of the 15-item IPARLS (Rohner and Molaver, 2015; Rohner et al., 2020) was administered to each participant. Items were scored on a 5-point Likert scale (from 1 = “never true” to 5 = “always”).

##### De Jong Gierveld Loneliness Scale (DJG-LS)

The Italian 6-item version of the DJG-LS (Van Tilburg et al., 1998; De Jong Gierveld and Van Tilburg, 2006) was administered to test the convergent validity of the IPARLS. The 6-item DJG-LS has proved to be a valid and reliable measurement instrument for overall, emotional (3-item), and social (3-item) loneliness. In the current study, the DJG-LS was administered to a randomly selected subsample of participants (*n* = 485). Two total scores were computed: the emotional score (DJG-LS-E) and the social score (DJG-LS-S). The reliability indices of the scale, measured by means of Cronbach's alpha and omega (ωt; Revelle and Condon, 2019), were 0.781 and 0.786 for the emotional dimension and 0.895 and 0.897 for the social dimension.

##### Lubben Social Network Scale

The LSNS (Lubben et al., 2006) was administered to have an objective measure of the social network to test the discriminant validity of the IPARLS scores. The scale assesses the richness of social networks of individuals, including family members and friends. In the current study, the scale was adapted to Italian, using standard forward- and backward-translation procedures (Van de Vijver and Tanzer, 2004), and administered to participants to test the divergent validity of the IPARLS. The LSNS was administered to a randomly selected subsample of participants (*n* = 203). Two total scores were computed: the family network score (LSNS-F) and the network score of peers (LSNS-P). The reliability indices of the scales, measured by means of Cronbach's alpha and omega (ωt), were 0.726 and 0.862 for the family network (LSNS-F) and 0.843 and 0.898 for the network of peers (LSNS-P).

#### Data Analyses

Preliminarily, univariate distributions of responses to each item were examined to verify missing data and normality (Shapiro and Wilk, 1965). The analysis of the missing data showed percentages of <1% on IPARLS items and on the other variables; therefore, for each analysis, in case of missing data, the relative units were excluded. Item scores showed a relevant deviation from normality for several IPARLS items. Therefore, the psychometric properties of the scale were investigated by means of robust statistics. In particular, the factorial structure of the IPARLS, the MI of the scale (across gender and age), and the concurrent and divergent validity of the scale were investigated. Confirmatory factor analysis (CFA) and MI analysis were performed with LISREL 8.71 software. All other analyses were performed with R 3.6.1 software. If not otherwise specified, an alpha level of 0.05 was used for all statistical tests. All reported *p*-values are two-tailed.

##### Confirmatory Factor Analysis

A robust CFA was carried out to test the unidimensionality of the 15-item version of the scale. As for fit indices, we used the maximum likelihood Chi-square test (*ML*χ*2*) in combination with other statistics less affected by sample size (Kline, 2011): (a) the root mean square error of approximation index *(RMSEA*); (b) the comparative fit index (*CFI*); and (c) the non-normed fit index (*NNFI)*. For *ML*χ*2* test, values associated with *p* > 0.05 were considered well-fitting models; for the *RMSEA* index, values up to 0.08 or lower were considered good fitting models; for the *CFI* and the *NNFI* indices, values >0.90 were considered as an adequate fit of the model to the data. Finally, the difference in *ML*χ*2* statistics (*ML*χ*2*diff) and *CFI* (*CFI*diff) values were used to test the relative fit of nested models (Putnick and Bornstein, 2016).

##### Measurement Invariance

MI across gender and age was verified. In all invariance analyses, the one-factor model has shown that adequate fit indices in the CFA were taken as reference. Invariance was verified according to the guidelines in the literature (Putnick and Bornstein, 2016). In particular, configural, metric, scalar, and residual invariance were tested by comparing covariance matrices computed as a function of the considered factors. To test MI across gender, two groups were compared, males (*n* = 337) and females (*n* = 351), whereas to test MI across ages, three groups were compared, G1 (19–25 years, *n* = 252), G2 (26–45 years, *n* = 228), and G3 (46–69 years, *n* = 208). The three age groups were defined on the basis of percentiles in order to have sufficiently large groups to perform the invariance analysis. The robust *ML* method was used to estimate parameters, and the same goodness-of-fit statistics as in the *CFA* were considered to verify the invariance of the matrices.

##### Reliability

Reliability of the IPARLS was examined using both Cronbach's alpha and omega (ωt) for ordinal measures (Revelle and Condon, 2019).

##### Construct Validity

To evaluate the validity of the IPARLS, Pearson's correlation coefficients between the IPARLS total scores and the two subscales of the DJG-LS (convergent validity) and the two subscales of LSNS (discriminant validity) were computed. The Hommel's correction to the *p*-values of the correlation coefficients was applied to control the increase of type I error (Hommel, 1988).

##### Effect of Gender and Age on Loneliness

To investigate the effects of invariance factors (gender and age) on loneliness, a linear multiple regression analysis was carried out to test direct and moderated effects. The model included three predictors: gender (dummy coded: male = 1, female = 0), age (z-score), and the gender × age interaction. The IPARLS total score was the dependent variable. Moreover, to define reference values for interpreting the observed scores, based on the results of the regression analysis and of a *CI* of 90%, the mean values and the lower and upper expected values as a function of gender and age were identified. According to the results, values outside the interval indicate the presence of a significantly different degree of distress related to loneliness.

### Results

#### Confirmatory Factor Analysis

Results confirmed that the one-factor model had adequate fit indices, *RMSEA* = 0.073, 90% *CI* [0.07; 0.08], *CFI* = 0.989, *ML*χ^2^ (86, *N* = 688) = 1,092.06, *p* < 0.001. Results showed that all items had a saturation >0.52 and that items 1 and 2, items 3 and 4, items 6 and 10, and items 8 and 14 had correlated error terms (see Table 1).

**Table 1:** Standardized factor loadings of the 15-item Interpersonal Acceptance-Rejection Loneliness Scale.

**Item**	**F1**
1. I feel badly because I am isolated from others	0.753
2. I feel unhappy because I am left out	0.784
3. I feel sad because I don't have companionship	0.825
4. I have a sense of emptiness because I lack friends	0.840
5. I feel dejected because my circle of friends is too limited	0.801
6. I feel lonely	0.771
7. It hurts to be so alone	0.751
8. I wish I had more friends	0.721
9. I feel like reaching out to others so I won't feel so alone	0.801
10. I am distracted by feelings of loneliness	0.784
11. It bothers me that I am so isolated	0.858
12. I am unhappy because too many others view me with indifference	0.786
13. I am unhappy because I am not part of a social group	0.866
14. I wish I had as many friends as other people	0.793
15. I could really use the company of others	0.519

*Responses were collected on the following scale: “Almost Never True”; “Not Often True”; “Sometimes True”; “Often True”; “Almost Always True.”*

#### Measurement Invariance

MI analysis showed that the IPARLS is a fully invariant scale as a function of both gender and age (see Table 2).

**Table 2:** Measurement invariance analysis: multi-group hierarchical confirmatory factor analyses.

**Model^**a**^**	**Goodness-of-fit indices**					
	**RMSEA**	**CFI**	**ML**χ^2^****	**df**	**ML$\chi_{\mathrm{diff}}^{2}$**	**CFI_**diff**_**
**Gender**						
M1	0.071	0.989	1313.3^***^	172	–	–
M2	0.068	0.987	1400.9^***^	187	87.6^***^	0.002^b^
M3	0.076	0.986	1456.8^***^	201	55.9^***^	0.001^c^
M4	0.060	0.990	1603.9^***^	220	147.1^***^	−0.004^d^
**Age**						
M1	0.079	0.986	1739.7^***^	258	–	–
M2	0.089	0.981	1981.0^***^	288	241.3^***^	0.005^c^
M3	0.093	0.977	2099.9^***^	316	118.9^***^	0.004^d^
M4	0.078	0.982	2637.3^***^	354	537.4^***^	−0.005^e^

a *M1, one-factor configural invariance (CI); M2, one-factor CI and metric invariance (MI); M3, one-factor CI, MI, and scalar invariance (SI); M4, one-factor CI, MI, SI, and invariant uniqueness; Gender: Males n = 337, Females n = 351; Age: G1 [19–25 years] n = 252, G2 [26–45 years] n = 228, G3 [45–69 years] n = 208*.

*** *p < 0.001*.

b *The reference model is M1*.

c *The reference model is M2*.

d *The reference model is M3*.

#### Reliability

The IPARLS showed good levels of internal consistency, as indicated by robust alpha and omega statistics, α = 0.959 and ωt = 0.969.

#### Construct Validity

The validity analysis showed that the IPARLS scores correlated significantly and strongly with the emotional dimension of the DJG-LS and correlated significantly with the social dimension of the DJG-LS. As expected, the latter was weaker than the former, and the two correlations were significantly different, z-test = 5.81, *p* < 0.001. No significant correlations were observed with the two objective measures of the social network, the LSNS-F and the LSNS-P scales. Therefore, results indicated that the scale had adequate convergent and discriminant validity (see Table 3).

**Table 3:** Descriptive statistics and correlations matrix between IPARLS and considered criterion variables.

**Measure^**a**^**	**Criterion variables**				**M (SD)**
	**DJG-LS-E**	**DJG-LS-S**	**LSNS-F**	**LSNS-P**	
IPARLS	0.730^***^^b^	0.504^***^^b^	−0.077	−0.140	28.91 (11.83)

a *IPARLS, Interpersonal Acceptance-Rejection Loneliness Scale (N = 688); DJG-LS-E, emotional dimension of the De Jong Gierveld Loneliness Scale (n = 485); DJG-LS-S, social dimension of the De Jong Gierveld Loneliness Scale (n = 485); LSNS-F, family network score of the Lubben Social Network scale (n = 203); LSNS-P, peers' network score of the Lubben Social Network scale (n = 203)*.

***b *Hommel's corrected p-value < 0.001*.

#### Effect of Gender and Age on Loneliness

**Figure 1:**
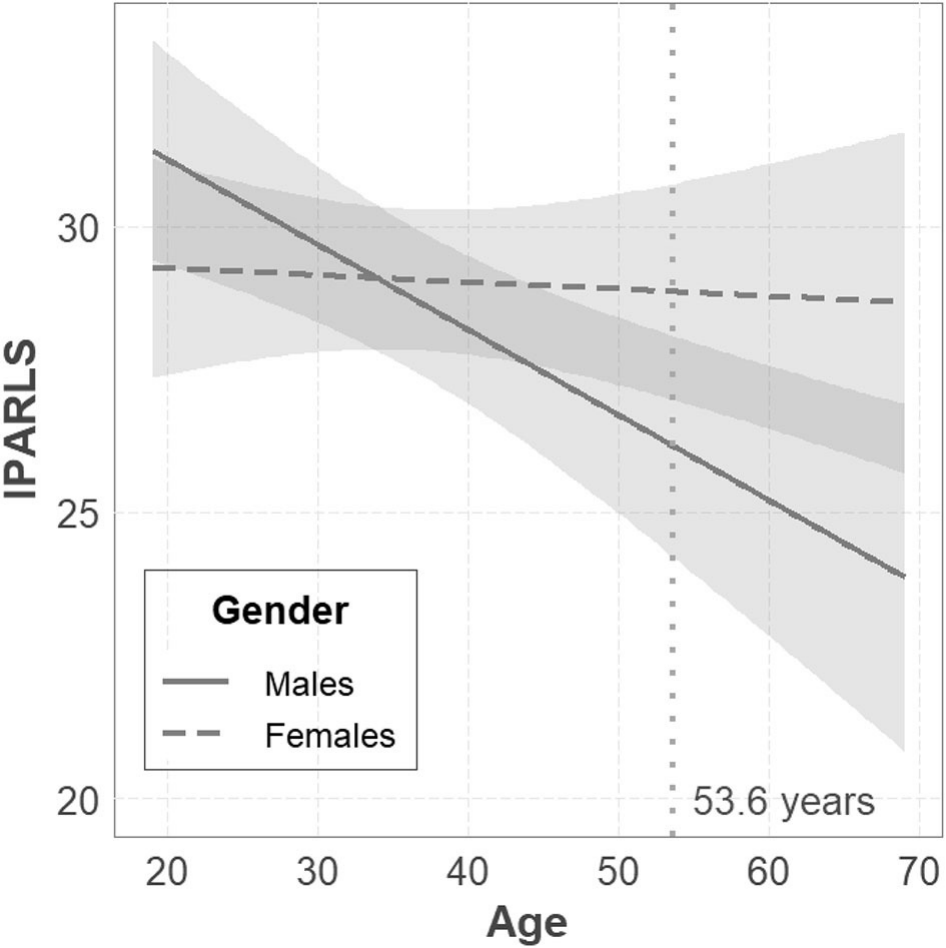
Moderation effect of gender on the relation between age and loneliness (IPARLS). Bandwidth indicates a confidence interval of 95%.

Results showed that the model, including gender and age as predictive factors, influenced perceived loneliness, *F*_(3, 684)_ = 4.091, *p* = 0.007, *R*^2^ = 0.018. In particular, results showed a significant gender × age interaction, *b* = −0.172, *p* = 0.024, indicating that, only for males, the greater the age, the lower was the perceived loneliness. No association between age and loneliness was observed in the female group (see Figure 1).

On the basis of the latter results, mean values and the 90% CIs with the lower and upper expected values as a function of gender and age were identified (see Table 4).

**Table 4:** Mean value and 90% CIs of expected loneliness distress as a function of gender and age.

**Age**	**Gender**	
	**Males**	**Females**
19	31 [12; 51]	29 [10; 49]
20	31 [12; 50]	29 [10; 49]
21	31 [12; 50]	29 [10; 49]
22	31 [12; 50]	29 [10; 49]
23	31 [11; 50]	29 [10; 49]
24	31 [11; 50]	29 [10; 49]
25	30 [11; 50]	29 [10; 49]
26	30 [11; 50]	29 [10; 49]
27	30 [11; 49]	29 [10; 49]
28	30 [11; 49]	29 [10; 49]
29	30 [11; 49]	29 [10; 48]
30	30 [10; 49]	29 [10; 48]
31	30 [10; 49]	29 [10; 48]
32	29 [10; 49]	29 [10; 48]
33	29 [10; 48]	29 [10; 48]
34	29 [10; 48]	29 [10; 48]
35	29 [10; 48]	29 [10; 48]
36	29 [10; 48]	29 [10; 48]
37	29 [9; 48]	29 [10; 48]
38	28 [9; 48]	29 [10; 48]
39	28 [9; 48]	29 [10; 48]
40	28 [9; 47]	29 [10; 48]
41	28 [9; 47]	29 [10; 48]
42	28 [9; 47]	29 [10; 48]
43	28 [8; 47]	29 [10; 48]
44	28 [8; 47]	29 [10; 48]
45	27 [8; 47]	29 [10; 48]
46	27 [8; 47]	29 [10; 48]
47	27 [8; 46]	29 [10; 48]
48	27 [8; 46]	29 [10; 48]
49	27 [8; 46]	29 [10; 48]
50	27 [7; 46]	29 [10; 48]
51	27 [7; 46]	29 [10; 48]
52	26 [7; 46]	29 [10; 48]
53	26 [7; 45]	29 [10; 48]
54	26 [7; 45]	29 [10; 48]
55	26 [7; 45]	29 [10; 48]
56	26 [7; 45]	29 [10; 48]
57	26 [6; 45]	29 [10; 48]
58	25 [6; 45]	29 [10; 48]
59	25 [6; 45]	29 [10; 48]
60	25 [6; 44]	29 [10; 48]
61	25 [6; 44]	29 [10; 48]
62	25 [6; 44]	29 [10; 48]
63	25 [5; 44]	29 [10; 48]
64	25 [5; 44]	29 [10; 48]
65	24 [5; 44]	29 [10; 48]
66	24 [5; 44]	29 [10; 48]
67	24 [5; 43]	29 [10; 48]
68	24 [5; 43]	29 [10; 48]
69	24 [5; 43]	29 [10; 48]

*Observed total scores outside the relative intervals indicate the presence of a significantly different degree of distress related to loneliness*.

### Discussion

Results confirmed that the scale has a unidimensional structure, adequate reliability, and adequate validity (Rohner and Molaver, 2015; Rohner et al., 2020). Moreover, for the first time, the analysis of the invariance (Putnick and Bornstein, 2016) of the scale was carried out considering gender and age factors. Data confirmed that the scale is fully invariant and it allows a valid gender and age differences evaluation.

The results of the construct validity analysis confirmed that IPARLS can accurately measure the emotional component of loneliness (Rohner and Molaver, 2015) and, more importantly, clarify that the scale measures subjective aspects rather than the richness of the social network (Lubben et al., 2006). Having verified the invariance of the scale across gender and age factors (Putnick and Bornstein, 2016), this study highlighted, in a valid way, that age is correlated with distress related to loneliness in males but not in females. This latter result is in line with that reported by Maes et al. (2019) and seems to better clarify gender differences and the effect of age on loneliness observed in the literature (Barreto et al., 2020; Shovestul et al., 2020). More studies are needed to confirm the robustness of this “small” moderation effect.

## Study 2

The second study aimed to further test the validity of the IPARLS by investigating the relationship between loneliness scores and the general psychological adjustment. Indeed, given that the previous literature shows that there is a strong correlation between loneliness and psychological adjustment (Heinrich and Gullone, 2006; Richardson et al., 2017; Rohner et al., 2019; Menec et al., 2020; Wang et al., 2020), the specific aim of this study was to investigate whether the IPARLS scores were correlated with psychological adjustment (concurrent validity) and whether the association is observed when controlling for gender and age differences. To this aim, the Italian version of IPARLS and the PAQ-SF (Rohner and Khaleque, 2012; Rohner and Ali, 2016; Rohner et al., 2019) were administered to a sample of 205 adults. Consistent with international literature, we expected to find a significant and specific association between loneliness and the general psychological adjustment.

### Methods

#### Participants

A sample of 205 adults living in different cities of the Campania region aged between 20 and 69 years (103 females, 101 males; *M* age = 44.7, *SD* = 14.2) participated in the study. The participants were recruited by convenience sampling. Similar to Study 1, the sample was heterogeneous in terms of the educational level, which ranged from “less than middle school” to “college and above” (Mdn = “partial college, at least 1 year of specialized training”), and the occupational level, which ranged from “higher executive, proprietor of large businesses, major professional, and others” to “no regular occupation” (Mdn = “smaller business owners, skilled manual laborers, craftsmen, tenant farmers, and others”). The considered measures were administered in-person and in a paper-and-pencil format. The average time to complete the protocol was about 18 min. Data were collected in conformity with the Declaration of Helsinki and the local Ethics Committee requirements. All participants signed a written informed consent before starting data collection.

#### Procedure and Measures

Participants completed a protocol consisting of three sections: (1) the sociodemographic form; (2) the IPARLS; and (3) the PAQ-SF.

##### Sociodemographics

All participants completed a questionnaire to collect socio-demographic information (age, gender, educational level, and so on).

##### Interpersonal Acceptance–Rejection Loneliness Scale

The Italian version of the 15-item IPARLS (Rohner and Molaver, 2015; Rohner et al., 2020) validated into Study 1 was administered to each participant. Items were scored on a 5-point Likert scale (from 1 = “never true” to 5 = “always”). A total score of distress related to loneliness was computed. The reliability indices of the scale, estimated by means of ordinal Cronbach's alpha and omega (ωt; Revelle and Condon, 2019), were 0.950 and 0.965.

##### Personality Assessment Questionnaire Short-Form

The Italian PAQ-SF (Rohner and Khaleque, 2012; Rohner and Ali, 2016; Rohner et al., 2019; Senese et al., 2020a) was administered to each participant to assess the general psychological adjustment (Rohner, 2004, 2019; Rohner and Lansford, 2017). The Adult PAQ-SF is a 42-item self-report questionnaire measuring perceptions of individuals about themselves with respect to seven dimensions: (1) hostility/aggression (6 items), that measures physical, verbal, and passive aggression and problems with the management of hostility and aggression; (2) dependence or defensive independence (6 items), that measures the psychological need for emotional support, care, comfort, attention, and nurturance and similar responses from significant others; (3) negative self-esteem (6 items), that measures the negative feelings of disliking or disapproving of oneself or perceiving oneself to be a worthless person or worthy of condemnation; (4) negative self-adequacy (6 items), that measures the negative feelings of incompetence, or perceived inability to meet day-to-day demands successfully; (5) emotional unresponsiveness (6 items), that measures the inability to express emotions freely and openly to others; (6) emotional instability (6 items), that measures the inability to control frequent and often unpredictable mood shifts that may swing from pole to pole; and (7) negative worldview (6 items), that measures the feeling that life is essentially bad, insecure, threatening, unpleasant, hostile, uncertain, and/or full of many dangers. For each item, individuals are asked to indicate the extent to which they think that each sentence is true for them on a 4-point Likert-type scale (from 4 = “almost always true of me” to 1 = “almost never true of me”). Higher scores on all scales indicate less positive psychological adjustment. In line with recent studies (Rohner et al., 2019, 2020; Senese et al., 2020a), a total score of psychological maladjustment was calculated for each participant by summing all the scales except for the “dependence or defensive independence” dimension, which was not correlated with the other subscales. The reliability indices of the maladjustment score, measured by means of ordinal Cronbach's alpha and omega (ωt; Revelle and Condon, 2019), were 0.939 and 0.952.

#### Data Analyses

Preliminary descriptive analyses were executed to investigate missing values and variable distributions. A descriptive analysis was conducted to calculate the basic statistics for age, gender, loneliness, and psychological maladjustment.

To investigate the association between the considered variables (age, gender, loneliness, and psychological maladjustment), Pearson's correlation coefficients were computed first. For each correlation coefficient, the Hommel's correction to *p*-values was also applied to control the increase of type I error (Hommel, 1988). Moreover, to investigate the specific association between loneliness and psychological maladjustment, a hierarchical multiple regression analysis was carried out. In this latter analysis, the psychological maladjustment was regressed on age, gender (as control variables), and loneliness. In the regression analysis, age and loneliness were included as z-scores, whereas the gender was dummy coded (males = 0; females = 1). In the first step, age and gender were included; in the second and final step, loneliness was added. All analyses were performed with the software R version 4.0.2 (R Core Team, 2020).

### Results

Correlation analyses (see Table 5) showed that psychological maladjustment was weakly and significantly associated with age, *r* = −0.16, *p* = 0.02, but strongly associated with loneliness, *r* = 0.53, *p* < 0.001. That is, the greater the age, the lower was the self-reported psychological maladjustment, whereas the greater the perceived loneliness, the greater was the self-reported psychological maladjustment. The latter association remained significant even when correcting the *p*-value by the Hommel's correction. The association between gender and psychological maladjustment was not significant.

**Table 5:** Descriptive statistics and Pearson's correlations.

**^**a**^Variable**	***M* (*SD*)**	**Skewness**	**Kurtosis**	**Correlations**		
				**1**	**2**	**3**
1. Gender	–	–	–	–		
2. Age	44.7 (14.2)	−0.1	−1.2	−0.02	–	
3. IPARLS	25.9 (9.8)	1.1	0.8	0.10	0.03	–
4. PAQ-SF	69.6 (15.4)	0.3	−0.3	0.10	−0.16^*^	0.53^***^^b^

a *Gender: gender of participants (dummy coded: males = 0; females = 1); Age, age of participants (years); IPARLS, Interpersonal Acceptance-Rejection Loneliness Scale; PAQ-SF, Personality Assessment Questionnaire short-form total score (higher scores indicate less positive psychological adjustment)*.

* *p-value < 0.05*.

*** *p-value < 0.001*.

b *Hommel's corrected p-value < 0.001*.

The results of hierarchical regression (see Table 6) confirmed the positive and strong association between loneliness and psychological maladjustment, β = 0.53, *p* < 0.001, showing that, over and above gender and age differences, loneliness and psychological maladjustment shared about 28% of the variance. Moreover, results showed that the association between age and psychological maladjustment was observed independently of gender and perceived loneliness, β = −0.170, *p* < 0.01.

**Table 6:** Hierarchical multiple regression analyses predicting psychological maladjustment from gender, age and loneliness (IPARLS; *N* = 204).

**Predictor^**a**^**	$R_{\mathrm{diff}}^{2}$	***B***	**β**
Step 1	0.034^*^^b^		
Gender		0.189	0.095
Age (z-score)		−0.156^*^	−0.156^*^
Step 2	0.279^***^		
Gender		0.083	0.041
Age (z-score)		−0.170^**^	−0.170^**^
IPARLS (z-score)		0.531^***^	0.531^***^
Total R^2^	0.313^***^		

a *Gender: gender of participants (dummy coded: males = 0; females = 1); Age, age of participants (years); IPARLS, Interpersonal Acceptance-Rejection Loneliness Scale; PAQ-SF, Personality Assessment Questionnaire short-form total score (higher scores indicate less positive psychological adjustment)*.

* *p < 0.05*.

** *p < 0.01*.

*** *p < 0.001*.

### Discussion

Results of Study 2 showed, as expected, that the perception of loneliness in adults is strongly associated with the general psychological adjustment and that this effect is observed beyond age and gender differences (Rohner et al., 2020). These results allowed further verification of the validity of the IPARLS scores (concurrent validity) and confirmed that the IPARLS is a valid tool for monitoring loneliness distress among the adult age groups.

## General Discussion

Based on the assumptions of IPARTheory, this study focuses on the investigation of the psychometric properties of the Italian version of the IPARLS (Rohner et al., 2020) that was adapted by Rohner and Molaver (2015) to overcome some limitations of the loneliness scales available in the literature and to measure the psychological distress associated with the feeling of loneliness. Specifically, considering that loneliness scales available in Italian do not provide complete indications on the psychometric properties; that in no case the invariance of the measure across gender and age has been verified; that the emotional and interpersonal component of loneliness is the one that most influences the well-being and adaptation of individuals, the aims of this work were 2-fold: (a) investigate the dimensionality, reliability, validity, and the MI of the scale across gender and age (Study 1); and (b) further testing the criterion validity (concurrent validity) of the scale by investigating the relationship between IPARLS scores of loneliness and the general psychological adjustment (Study 2).

Study 1 confirmed that IPARLS is a unidimensional, reliable, valid, and across age and gender fully invariant measure of loneliness (Putnick and Bornstein, 2016). Results showed that IPARLS measures mainly the emotional component of loneliness (Weiss, 1973), that is the subjective feelings about the psychological distress associated with interpersonal loneliness. Moreover, the invariance analysis indicated that the scale can be validly used to compare males and females and adults aged between 19 and 69 years. Finally, results showed a “small” moderation effect of the gender on the association between age and loneliness distress, indicating that only for males the greater the age the lower the reported loneliness (see Barreto et al., 2020; Shovestul et al., 2020). These results are in line with those reported in the recent meta-analysis investigating the relationship between gender, age, and loneliness (Maes et al., 2019). Furthermore, it is important to emphasize that these results are particularly significant because the comparability of loneliness scores by gender and age was previously verified with the invariance analyses (Putnick and Bornstein, 2016). It would be useful to replicate the results of this study to clarify whether the conflicting evidence in the literature may be related to limitations of the measures and not the factors considered.

Study 2, as expected, showed that IPARLS scores are positively and strongly related to psychological adjustment. The more the individuals feel distressed by loneliness, the higher their reported psychological maladjustment. This result is consistent with the previous literature (Heinrich and Gullone, 2006; Richardson et al., 2017; Menec et al., 2020; Rohner et al., 2020; Wang et al., 2020) highlighting the link between loneliness, psychosocial difficulties, and mental health and is consistent with the IPARTheory, postulating that the perception of unsatisfactory and rejecting interpersonal relationships are associated with psychological maladjustment (Rohner, 2019).

This study has several strengths and limitations that should be noted. In terms of the merits, this study: (a) confirmed the psychometric adequacy of the Italian version of IPARLS; (b) the validity of the IPARLS scores when tested for the first time showing that the scale has a good construct and criterion validity; (c) showed that the Italian version of IPARLS can be validly used to measure loneliness distress across gender and age, thus confirming that this scale can be used to monitor the level of distress in the life cycle; and (d) defined CIs that can be considered in the clinical practice as a reference to interpret the observed score of distress linked to loneliness. On the other hand, there are some limitations that should be noted. First, one limitation is the need to correlate the errors of some items in order to obtain an adequate fit of the unidimensional latent model. The reason is that some items evaluate very similar aspects of the construct and the scale has no reversed item, thus not controlling the threat of response style (e.g., acquiescence, careless responding). A possible solution could be to eliminate redundant items by developing a short but equally valid version of the measure and/or to develop some new reversed items. We have not followed this direction in this study as it would mean changing the scale in a way that would no longer be comparable with the other IPARLS available versions. In the future, we could think of validating a short version of the instrument with some reverse items by directly testing its invariance across the different versions and evaluating the main factors influencing loneliness. Second, another limitation of the study is that the instrument does not allow us to define the extent to which the observed IPARLS scores are indicative of a pathological level of distress. In this study, we have suggested possible reference values as tentative to define mean values and 90% CI, indicating the lower and upper expected values as a function of gender and age. However, it is worth noticing that further studies are needed to identify validated clinical cutoffs, given the connection between loneliness distress and the different psychopathologies (Heinrich and Gullone, 2006; American Psychiatric Association, 2013; Richardson et al., 2017; Menec et al., 2020; Rohner et al., 2020; Wang et al., 2020). Third, the design of the study is correlational; therefore, we cannot be sure if it is loneliness that influences the psychological adjustment or *vice versa*; in other terms, the validity of the causal relationships is threatened. Further studies considering different methodologies (e.g., longitudinal, experimental) should be carried out to directly verify the direction of causality. Finally, another limitation is related to external validity. Indeed, in both studies, participants were recruited only from a region of southern Italy; this specificity could threaten the replicability of the results. Indeed, although the data indicated that the samples had heterogeneous characteristics in terms of gender, age, level of education, and occupational level, it is possible that loneliness is culturally influenced. Therefore, future studies should replicate the results by considering different samples.

In conclusion, despite its limitations, this study confirms that IPARLS is a valid instrument to investigate loneliness distress in the human life cycle, both from a clinical and a research point of view. This result may be of particular interest to those scholars interested in investigating if, and how, the introduction of artificial cognitive systems can be useful to increase the well-being of the elderly (Esposito et al., 2014; Baranyi et al., 2015). In fact, in our opinion, IPARLS will enable not only the identification of those who suffer most from loneliness to plan a target intervention but also the verification of whether the introduction of social agents or robots is efficient in reducing the levels of loneliness as hypothesized (Gallego-Perez et al., 2013; Gross et al., 2015).

## Data Availability Statement

The raw data supporting the conclusions of this article will be made available by the corresponding author, without undue reservation.

## Ethics Statement

Ethical approval was not provided for this study on human participants because data were collected in conformity with the Declaration of Helsinki and the Local Ethics Committee requirements. All participants signed a written informed consent before starting data collection.

## Author Contributions

VPS, CN, IS, and AG conceived the manuscript, wrote a part of the manuscript, and critically reviewed it. VPS supervised the data collection and analysis. CN and FM collaborated to the data analysis. FM and RM wrote a part of the manuscript, contributed to the intellectual contents, and critically reviewed it. All authors have approved the final manuscript.

## Conflict of Interest

The authors declare that the research was conducted in the absence of any commercial or financial relationships that could be construed as a potential conflict of interest.
